# Surface engineering of titania nanotubes incorporated with double-layered extracellular vesicles to modulate inflammation and osteogenesis

**DOI:** 10.1093/rb/rbab010

**Published:** 2021-05-11

**Authors:** Qingyu Zhao, Yi Zhang, Lan Xiao, Haiping Lu, Yaping Ma, Qi Liu, Xin Wang

**Affiliations:** 1 Department of Orthopaedic Surgery, Affiliated Hospital of Zunyi Medical University, Zunyi, 563003 Guizhou, China; 2 Department of Hygiene Toxicology, School of Public Health, Zunyi Medical University, Zunyi, 563000 Guizhou, China; 3 Institute of Health and Biomedical Innovation, Queensland University of Technology, 60 Musk Avenue, Kelvin Grove, Brisbane, QLD 4059, Australia; 4 The Australia−China Centre for Tissue Engineering and Regenerative Medicine (ACCTERM), Queensland University of Technology, Brisbane, QLD 4059, Australia; 5 Department of Periodontology, Stomatological Hospital of Zunyi Medical University, Zunyi, 563000 Guizhou, China

**Keywords:** EVs, TNT, hybrid, macrophages, MSCs, osteogenesis

## Abstract

Titania nanotubes (TNT) generated on titanium implant are emerged as important modification technique to facilitate bone regeneration. Mesenchymal stem cells (MSCs)-derived exosomes are membrane bound extracellular vesicles (EVs), which play an important role in tissue regeneration. The objective of this study was to generate an EVs hybrid TNT aiming at regulating inflammation, MSCs recruitment and osteogenesis. We isolated EVs from MSCs (MSCs EVs) and 3-day osteogenically differentiated MSCs (3d EVs). MSC EVs and 3d EVs exhibited round morphology under TEM, which also showed robust internalization by human bone marrow derived MSCs (hBMSCs). Next, we fabricated 3d EVs/MSC EVs hybrid TNT. When inflammatory macrophages were co-cultured with EVs hybrid TNT, the gene and protein expression of inflammatory cytokine were significantly reduced. Macrophage morphology was also examined by confocal laser scanning microscopy (CLSM) and scanning electron microscopy (SEM). Further migratory ability study using hBMSCs indicated significant enhancement of MSCs migration in EVs hybrid TNT. In addition, we further demonstrated significant increase of osteogenic differentiation of hBMSCs in EVs hybrid TNT. This study suggests that EVs hybrid TNT may serve as a viable therapeutic approach to enhance osteogenesis and bone regeneration.

## Introduction

Total joint and knee arthroplasty are one of the most common and cost-effective orthopedic surgeries for patients who suffer from joint diseases like osteoarthritis. The number of patients required such surgical management has increased substantially due to the aging society, thus causing huge social and economic burdens in recent years. It is predicated that the number of total knee replacements will reach 1.37 million in the US alone by 2020 [1], with the majority of patients were above the age of 45 [2]. In other developing countries, like China, the demand for joint replacement surgeries is continuously rising approximately 15% annually due to ever-increasing aging population [3].

The past decades have witnessed unprecedented growth in demands for novel scaffolds to meet the requirement of orthopedic surgeries [4]. Titanium is one the most important and widely used materials for orthopedic surgeons due to its excellent mechanical property and biocompatibility. Over the past decades, several strategies have been applied to modify the titanium surface, either physical or chemical, in order to enhance the performance and osseointegration of titanium implants for various applications. For instance, generation of well-aligned titanium dioxide (TiO_2_) nanotubes (TNT) has received increasing attention due to their unique functionality [5]. Previous study indicated that cellular behavior, including cellular migration, proliferation, and differentiation, could be affected by the topographies and structures of TNT [6]. For instance, one of the first studies indicated that 20 nm TNT offered the best environment for cell adhesion, migration, and proliferation in glioma and osteosarcoma cells [7]. In addition, mesenchymal stem cells (MSCs) grown on different sized TNT indicated that larger diameter TNT can significantly promote cell elongation and differentiation compared to smaller nanotubes [8]. Several attempts have also been made to incorporate bioactive molecules, like growth factors or osteoinductive molecules, inside the TNT in order to increase its bio-functionality and biocompatibility [9].

MSCs are a population of stem cells possess self-renewal and multiple lineages differentiation potential [10]. In recent years, several studies have indicated the important regenerative potential of bioactive factors released from MSCs including exosomes [11]. Exosomes belong to a group of extracellular vesicles (EVs) secreted by cells, which play an important role in cellular communication. Exosomes from MSCs represent one of the most widely employed cell-free based therapies for tissue regeneration. Exosomes from MSCs hold multiple regenerative abilities, including reducing myocardial ischaemia-reperfusion injury [12], restoring renal injury and dysfunction [13], ameliorating lung injury [14], etc. In addition, MSCs-derived exosomes can significantly promote bone regeneration. For example, in a rat calvaria bone defect model, MSC-derived exosomes could significantly promote *in vivo* bone healing and angiogenesis [15]. One study using radiation-induced bone loss model also revealed that immediate intravenous injection of MSC-derived exosomes to radiation-exposed animals offer significant protective effect against bone loss, which the result is similar to MSC-based therapy [16]. In recent years, scaffolds with the incorporation of exosomes are getting widely attractions among researchers due to the easy-to-handle nature and important regulatory property of exosomes than their mother cells, especially biomaterial with limited immune-regulatory properties. For instance, incorporation of exosomes from Bone morphogenetic protein-2 (BMP-2) stimulated macrophages into TNT was fabricated previously, demonstrating significant pro-osteogenic effect on MSCs differentiation [17]. Another study also indicated cell adhesion and proliferation promoting ability for MSCs-derived exosomes immobilized titanium surface, indicating beneficial effect of exosome coating on cellular behaviors [18].

During bone regeneration, attraction and recruitment of adequate number of circulating progenitor cells or endogenous MSCs to the site of injury are very important for successful bone repair [19]. Therefore, enhancing the migration of MSCs has become one of the therapeutic strategies in bone regenerative medicine. Scaffolds incorporating various cytokines and chemokines that serve as MSCs attractant have been developed, with the aim of modulating MSCs response and reinforcing the mobilization of MSCs [20, 21]. Previous study also indicated that during osteogenic differentiation, exosomes isolated from 3-day osteogenically differentiated MSC have significant migratory promoting potential for naïve MSCs [22]. Therefore, we firstly isolated and characterized MSC-derived exosomes (MSC EVs) and 3-day osteogenically differentiation-derived exosomes (3d EVs). Poly(dopamine) coating is one of the most commonly used techniques for implant surface modification due to its unique adhesive properties [23]. Next, we generated first bioactive EVs (MSC EVs) layer using poly(dopamine) coating strategy. As a hydrogel with biocompatible and biodegradable properties, carboxymethyl chitosan hydrogel was used to fabricate second bioactive EVs (3d EVs) layer [23]. In addition, macrophages represent one of the principal responders to biomaterials and major mediator of wound healing [24]. Therefore, we also tried to investigate the immunoregulatory role of this EVs hybrid scaffold on macrophages and its subsequent impact on osteogenesis. Generating EVs hybrid TNT may provide a novel concept for other EVs inspired scaffolds.

## Materials and methods

### Reagents

Exosome isolation reagent (from cell culture media) was purchased from Thermo Fisher scientific (4478359, China). PKH67 Green Fluorescent Cell Linker Kit for General Cell Membrane Labeling (MINI67-1KT, China) and PKH26 Red Fluorescent Cell Linker Kit for General Cell Membrane Labeling (PKH26GL-1KT, China) were purchased from Sigma. Lipopolysaccharide (LPS, Escherichia coli 0111: B4, L4391), β‐glycerophosphate (G9422), L‐ascorbic acid 2‐phosphate (49752), and dexamethasone (D1756) were from Sigma, China. Antibodies used in this study were rabbit polyclonal antibody against alkaline phosphatase (ALP) (ab108337, Abcam), CD63 Antibody (sc-5275, SANTA CRUZ Biotechnology), CD81 Antibody (sc-166029, SANTA CRUZ Biotechnology), calnexin (2679 T, cell Signaling Technology), Grp94 (20292 T, cell Signaling Technology), and Lamin A/C (4777 T, cell Signaling Technology). Secondary antibody used for immunofluorescence staining was fluorescein isothiocyanate (FITC)-conjugated goat anti-rabbit IgG (H + L) Secondary Antibody (31635, Thermo Fisher Scientific, China).

### Cell culture

The murine-derived macrophage cell line RAW 264.7 cells (ATCC^®^ TIB-71™), and human bone marrow-derived mesenchymal stromal cells (hBMSCs; ATCC^®^ PCS-500-012™) were used for the study. RAW 264.7 cells were maintained in Dulbecco’s Modified Eagle’s Medium (DMEM; Life Technologies Pty Ltd., China) supplemented with 10% heat inactivated fetal bovine serum (FBS; Biological Industries, LTD, Beit Haemek, Israel), 1% (v/v) penicillin/streptomycin (Solarbio, Beijing, China) in a humidified incubator containing 5% CO_2_ at 37°C. hBMSCs were maintained in DMEM containing 10% of FBS and supplemented with 1% (v/v) of a penicillin/streptomycin solution.

### Cell culture-conditioned medium collection and EVs isolation

For hBMSCs-derived EVs isolation, confluent hBMSCs were cultured with serum-free DMEM for 24 h. hBMSCs-derived conditioned medium was collected [25], pooled and stored at –80 °C immediately until EVs isolation. For 3d-EVs isolation, confluent hBMSCs were cultured with DMEM containing 10% of FBS and supplemented with osteogenic components (2 mM β-glycerophosphate, 100 μM l-ascorbic acid 2-phosphate, and 10 nM dexamethasone). At 3 days, the 10% FBS supplemented DMEM was completely removed and replaced by serum-free DMEM as described previously [22, 26]. 3d-derived conditioned medium was collected, pooled and stored at –80 °C immediately until EVs isolation. EVs were isolated using exosome isolation reagent (from cell culture media) according to the manufacturer’s instructions. Briefly, the cell culture-conditioned medium was centrifuged at 2000*g* for 30 minutes to remove cells and debris. The supernatant was transferred to a new tube without disturbing the cells and debris pellet. One-half volumes of the total exosome isolation reagent were added into the supernatant before vortexing thoroughly. After overnight incubation at 4 °C, the mixture was centrifugated at 10 000*g* for 1 h at 4 °C to pellet exosomes. After removal of the supernatant, exosomes were resuspended in 50 μL of phosphate-buffered saline (PBS), aliquoted, and stored at –80°C immediately until further analysis.

### Transmission electron microscopy (TEM)

TEM was used to visualize the EVs isolated in this study. In brief, carbon/formvar-coated Cu TEM grids were placed on 5 μL EVs solution for 10 min. After removing EVs solution, the grids were stained with 1% uranyl acetate (UA, AGR1260A; Agar Scientific, China) for 10 s before washing three times with deionized water for 20 s. The grids were dried by Whatman filter paper. TEM images were taken by TEM (JEM-1400, JOEL, Japan) at 80 kV.

### Nanoparticle tracking analysis (NTA)

The size of EVs were characterized using Nanoparticle tracking analysis of Malvern NanoSight NS300 system (Malvern Instruments, United Kingdom). Briefly, EVs diluted in PBS (1:500) were loaded into a 1 mL syringes (BD Discardit II, New Jersey, USA) before automatic infusion. EVs infusion into the sample chamber was performed at room temperature with a flow rate of 20 µL/s for all experiments. Five 60 s videos were captured for each EV, and the EV sizes were analyzed by Nanoparticle tracking analysis software.

### Western blot

Exosomal protein concentration was determined using the Pierce™ BCA Protein Assay Kit (Thermo Fisher Scientific, China). Exosomes (20 µg in terms of protein) were suspended in RIPA lysis buffer (89901, Thermo Fisher Scientific, China). Proteins were separated using 4–12% gradient Bolt™ Bis-Tris Plus Gel (Thermo Fisher Scientific, China), transferred to polyvinylidene difluoride (PVDF) membranes, and blotted with antibodies against CD63 (1:100) or CD81 (1:100). Non-exosomal markers calnexin (1:1000), Grp94 (1:1000), and Lamin A/C (1:1000) were used as negative control. After washing, the blots were incubated with IRDye^®^ 800CW goat anti-mouse IgG (1:10 000; LI-COR Biotechnology, USA) or IRDye^®^ 800CW goat anti-rabbit IgG (1:10 000; LI-COR Biotechnology, USA) and washed thrice with PBS-Tween 20 (0.1%). Protein signals were visualized using Odyssey infrared Imaging System (LI-COR Biotechnology, USA).

### EVs labeling and examination

EVs internalization into recipient cells were further characterized using PKH26 red Fluorescent Cell Linker Kit for General Cell Membrane Labeling according to the manufacturer's instructions. In brief, 500 μL Diluent C was used to dilute EVs before mixing with PKH26 dye suspended in 500 μL of diluent C for 5 min. About 1 mL of DMEM with 1% exosome‐depleted FBS (A2720803; Thermo Fisher scientific, China) was used to stop the staining process as previously described [22]. Equal volume of DMEM with 10% exosome‐depleted FBS was added before incubating with hBMSCs for 12 h at 37 °C. Samples were fixed by 4% paraformaldehyde (PFA). Cells were photographed using an inverted confocal laser scanning microscope (Leica DM IRB; Leica, Wetzlar, Germany).

### Growth of titania nanotubes and EVs hybrid titania nanotubes

Commercial pure titanium discs (0.89 mm thickness, 99.7% purity; Alfa-Aesar, Ward Hill, MA, USA) were used in this study. Titanium discs were mechanically polished before ultrasonically cleaned in acetone, ethanol, and deionized water for 10 minutes. Electrochemical anodization was used to fabricate TNT. Titanium discs were anodized in an ethylene glycol (EG) solution containing 0.3 wt% ammonium fluoride and 5 vol% H_2_O at 30 V for 1 h. Subsequently, the samples were washed immediately after anodization using deionized water and air dried. For dopamine coating, TNT were soaked in Tris–HCL buffer solutions (10 mM, pH 8.5) with 2 mg/mL of dopamine hydrochloride (H8502; Sigma, China) for 24 h in darkness with continuous stirring. After rinsing with deionized water, TNT were dried before MSC EVs deposition. Exosomes (10 µg in terms of protein) were used according to previous study [27]. For MSC EVs deposition, MSC EVs (10 μg protein equivalent) diluted in PBS were incubated with TNT for 1 h. Carboxymethyl chitosan hydrogel layer was generated as previously described [23]. In brief, 50 μL carboxymethyl chitosan and genipin solution were mixed with 3d EVs (10 μg protein equivalent) and dropped onto the surface and dried.

To determine exosome deposition on different layers, different exosome-labeling markers were used as described previously [17]. In brief, MSC EVs were prelabeled with green labeling marker, PKH67, while 3d EVs were prelabeled with red labeling marker PKH26. Different layers of EVs coating were performed as mentioned above.

### MTT assay

The metabolic activity of macrophages cultured on different scaffolds was evaluated using MTT [3-(4, 5-dimethylthiazol-2-yl)-2, 5-diphenyl tetrazolium bromide] assay (M2128; Sigma, China). In brief, 20 µL of 5 mg/mL MTT solution was added into each well for 4 h. After carefully removing the supernatant, 100 µL dimethyl sulfoxide (DMSO) was used to dissolve the formazan crystals. The absorbance was read on microplate reader (Benchmark Plus, USA) at 490 nm.

### Inflammatory macrophage response to different scaffolds

To determine the effect of inflammatory macrophages response to different scaffolds, co-culture study was performed. In brief, macrophages were seeded on coverslip at a density of 1 × 10^5^ overnight at 37°C before stimulating with 1000 ng/mL of LPS. After 6 h stimulation, LPS was removed, cells were rinsed trice with PBS before co-culturing with different scaffolds. After 24 h of incubation, cells were fixed by 4% PFA and stained with Alexa Fluor 594-labeled phalloidin. Cells were photographed using an inverted confocal laser scanning microscope (Leica DM IRB; Leica, Wetzlar, Germany). iNOS expression was examined as previously described [22]. In brief, cells were fixed, permeabilized, and blocked before incubating with a rabbit polyclonal antibody against iNOS (ab3523; Abcam). Actin was stained with Alexa Fluor 594-labeled phalloidin. Images were taken using confocal laser scanning microscope (Leica DM IRB; Leica, Wetzlar, Germany).

### Scanning electron microscopy (SEM)

To further investigate the morphology of inflammatory macrophages response to different scaffolds, SEM was used to image cells. In brief, samples were washed by PBS at least three times, and then fixed in 3% glutaraldehyde in 0.1 M sodium cacodylate buffer (pH 7.4) overnight at 4°C. After rinsing three times with 0.1 M sodium cacodylate buffer (10 min each), the samples were post-fixed with 1% osmium tetroxide (OsO_4_), and dehydrated using 50%, 70%, 90%, and 100% ethanol. The samples were dried by hexamethyldisilazane (HMDS) solution, then mounted on carbon tabs and sputter coated with gold-palladium. All specimens were analyzed under a Zeiss SEM (Carl Zeiss NTS, Germany).

### RNA extraction and real-time quantitative polymerase chain reaction (RT-PCR)

Cells were firstly lysed using Trizol reagent (15596026; Thermo Fisher Scientific, China). Total RNA was then isolated using PureLink™ RNA Mini Kit (12183018 A; Thermo Fisher Scientific, China) according to the manufacturer's instructions. RNA concentration was measured using NanoDrop 8000 spectrophotometer (NanoDrop technologies). cDNA synthesis was performed using the RevertAid First Strand cDNA Synthesis Kit (K1622; Thermo Fisher Scientific, China) according to manufacturer’s instruction. About 500 ng of total RNA was used as template for reverse transcription. RT-PCR was performed using SYBR Green qPCR Master Mix (Life Technologies, China) on an ABI Prism 7500 Thermal Cycler (Applied Biosystems, Foster City, California, USA). All primer sequences (Table 1) were analyzed for target specificity on Primer BLAST and were purchased from Sigma, China. The mRNA expression of the genes of interest was normalized against the housekeeping gene GAPDH. The difference between the mean Ct values of the gene of interest and the housekeeping gene was labeled ΔCt and the relative expression was calculated using the comparative Ct (2^−ΔΔCT^) method [28].

**Table 1.** Primers used in RT-PCR

Genes	Primer sequences
IL-6	Forward: 5′-ATAGTCCTTCCTACCCCAATTTCC-3′ Reverse: 5′-GATGAATTGGATGGTCTTGGTCC-3′
iNOS	Forward: 5′-CGAAACGCTTCACTTCCAA-3′ Reverse: 5′- TGAGCCTATATTGCTGTGGCT-3′
TNF-α	Forward: 5′-CTGAACTTCGGGGTGATCGG-3′ Reverse: 5′-GGCTTGTCACTCGAATTTTGAGA-3′
IL-10	Forward : 5'- GAGAA CATGGCCCAGAAATC-3′ Reverse : 5′- GAGAAATCGATGACAGCGCC-3′
CD206	Forward: 5′- AGACGAAATCCCTGCTACTG -3′ Reverse: 5′- CACCCATTCGAAGGCATTC -3′
Murine GAPDH	Forward: 5′-TGACCACAGTCCATGCCATC-3′ Reverse: 5′-GACGGACACATTGGGGGTAG-3′
ALP	Forward: 5′-CGTGGCTAAGAATGTCATCATGTT-3′ Reverse: 5′-TGGTGGAGCTGACCCTTGA-3′
Col-I	Forward: 5′-CCCTGGAAAGAATGGAGATGAT-3′ Reverse: 5′-ACCATCCAAACCACTGAAACCT-3′
Runx2	Forward: 5′-CATGGCGGGTAACGATGAA-3′ Reverse: 5′-AGACGGTTATGGTCAAGGTGAAA-3′
OPN	Forward : 5′- GACCAAGGAAAACTCACTAC-3′ Reverse: 5′- CTGTTTAACTGGTATGGCAC-3′
BMP-2	Forward: 5′- CTGAACTCCACTAATCATGC-3′ Reverse: 5′- CTGCCCTGTTACTGCCATTATT-3′
BMPRII	Forward: 5′- GGCAGCAGTATACAGATAGGTG-3′ Reverse: 5′- CTGCCCTGTTACTGCCATTATT-3′
Human GAPDH	Forward: 5′-TCAGCAATGCCTCCTGCAC -3′ Reverse: 5′-TCTGGGTGGCAGTGATGGC -3′

### Enzyme-linked immuno-sorbent assay (ELISA)

To further investigate the inflammatory macrophages response to different scaffolds, the level of inflammatory cytokine was measured using commercial ELISA kit (R&D Systems, China), following the manufacturer’s instructions. In brief, the culture media was collected and centrifuged at 500*g* for 10 min to remove cell debris. The cytokine concentrations were determined by correlation with a standard curve and the outcomes were expressed as the amount (pg) per milliliter of supernatants. Each experimental condition was assayed at least three times.

### Migratory ability of hBMSCs to different scaffolds

The migration assay was used to determine the effect of different scaffolds on hBMSCs’ migration. Briefly, a six-well transwell inserts with pores of 8.0 μm in size (Becton-Dickinson Labware, USA) was used for the migration assay. hBMSCs were placed in the upper chamber overnight at 37°C. Different scaffolds were deposited in the lower chamber overnight. Non-migrated cells in the upper chamber were gently removed using a cotton swab, and the migrated cells adhering to the lower chamber were fixed by 4% PFA and stained with Alexa Fluor 594-labeled phalloidin. Cells were photographed using an inverted confocal laser scanning microscope (Leica DM IRB, Leica, Wetzlar, Germany). The migrated cells were quantified in five randomly selected fields on each transwell membrane.

### hBMSCs response to EVs hybrid scaffolds

To determine the effect of hBMSCs response to different scaffolds, hBMSCs were cultured on different EVs hybrid scaffolds for 1 day and 3 days. Cells were fixed by 4% PFA and stained with Alexa Fluor 594-labeled phalloidin. Cells were photographed using an inverted confocal laser scanning microscope (Leica DM IRB, Leica, Wetzlar, Germany). For ALP immunofluorescence staining, hBMSCs seeded at a density of 1 × 10^5^ were co-cultured with different scaffolds for 7 days under osteogenic medium. Cells were fixed with 4% PFA for 10 min at room temperature. Cells were permeabilized using Triton X-100 for 5 min, blocked with 4% bovine serum albumin for 1 h at room temperature, and then incubated with ALP (1:100). After overnight incubation, cells were washed trice with PBS, then incubated with fluorescein isothiocyanate (FITC)-conjugated goat anti-rabbit IgG (H + L) secondary antibody. Actin was stained using Alexa Fluor 594-labeled phalloidin. Samples were prepared for observation using an inverted confocal laser scanning microscope with a 40 × 1.3 NA oil objective (Leica DM IRB, Leica, Wetzlar, Germany). For ALP activity assay, hBMSCs seeded at a density of 1 × 10^5^ were co-cultured with different scaffolds for 7 days under osteogenic medium. ALP activity was determined using Alkaline Phosphatase Assay Kit according to manufactures instruction. To analyze gene expression, hBMSCs seeded at a density of 1 × 10^5^ were cocultured with different scaffolds for 3 days in osteogenic medium. RT-PCR was performed to evaluate osteogenesis-related genes expression.

### Statistical analysis

Data from all the experiments were presented as mean ± standard deviation (SD), and comparisons were made using GraphPad Prism 8.0 between two groups using two-tailed student’s *t*-test, and between three groups using one-way analysis of variance (ANOVA), with a post-hoc test (Holm–Sidak’s tests). Differences were considered statistically significant if *P *<* *0.05.

## Results and discussion

### EVs isolation and characterization

**Figure 1.**
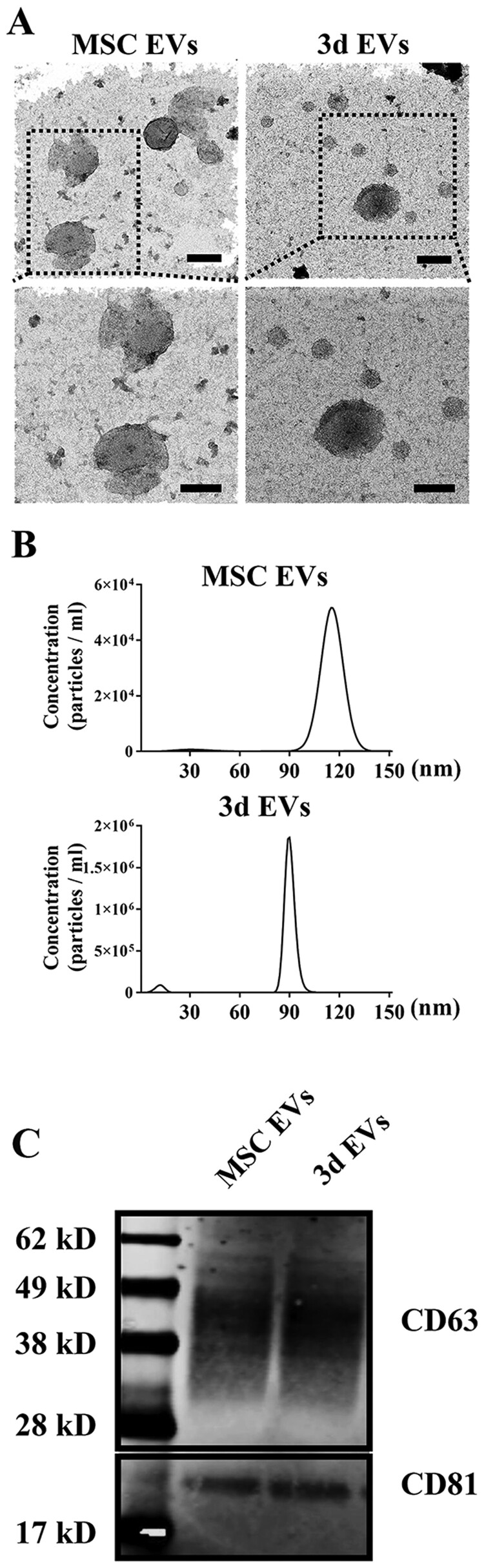
EVs isolation and characterization. (A) Transmission electron microscopy (TEM) micrographs of isolated EVs. Representative TEM images were taken from EVs isolated from hBMSCs (MSC EVs) and 3-day osteogenic differentially hBMSCs (3d EVs). Isolated EVs were negatively stained with 1% uranyl acetate (UA) before TEM imaging. Scale bar = 100 nm (top panel); 80 nm (lower panel). (B) Nanoparticle tracking analysis for isolated EVs. EVs were diluted in PBS (1:500) for nanoparticle tracking analysis. Representative size distribution and concentration of EVs isolated from hBMSCs and 3-day osteogenic differentially hBMSCs are shown. (C) Characterization of exosome markers (CD63 and CD81) by Western blot. Lane 1: protein marker; lane 2: MSC EVs; lane 3: 3d EVs.

MSCs from bone marrow stromal are one of the commonly used stem cells in the field of tissue regeneration. Several mechanisms have been proposed for MSCs induced tissue regeneration [29]. One of the most important mechanisms is by producing multiple secretory factors via paracrine mode [30]. The beneficial effect of cell culture-conditioned medium from MSCs on various diseases have been implicated. For example, in antigen-induced arthritis model, intra-articular injection of MSCs derived conditioned medium can significantly reduce knee-joint swelling and symptoms induced by arthritis [31]. In addition to various growth factors, exosomes represent one of the most important intracellular mediators. Exosomes are a group of nanoparticles with diameters approximately ranging from 40 to 100 nm. It seems that during osteogenic differentiation, exosomes releasing pattern or its microRNA profiles changes accordingly depending on the stage of their differentiation. Wang et al. [32] isolated a series of exosomes from hMSCs during osteogenic differentiation and found important microRNAs related to osteogenic differentiation from the late stage of differentiated hMSCs. Although the work by Wei et al. [22] indicated only exosomes isolated from MSCs holds pro-osteogenic effect, which is consistent with previous studies [33, 34]. This indicates the potential use of MSCs-derived exosomes as therapeutic approaches for bone/tissue regeneration. In this study, structural analysis of EVs from MSCs (MSC EVs) and 3d osteogenically differentiated MSCs (3d EVs) by TEM showed there was no significant difference between MSCs EVs and 3d EVs (Fig. 1A). In the MSCs-EVs and 3d-EVs groups, the EVs all exhibited round or cup-shaped morphology under TEM with a diameter around 100 nm. In addition, previous studies indicated that MSCs-derived exosomes exhibited similar appearance despite the cell origin. For example, human corneal mesenchymal stromal cell-derived exosomes exhibited round morphology with certain compression in the center [35]. EVs protein concentration was shown in Supplementary Fig. S1. In addition, MSCs EVs exhibited a slightly larger diameter (100.8 ± 14.7 nm) than the 3d EVs (90.4 ± 4.7 nm) as determined by nanoparticle tracking analysis (Fig. 1B). Western blot analysis further demonstrated the expression of exosomal marker proteins (CD63 and CD81) in lysates from the MSCs EVs and 3d EVs. In addition, calnexin, Grp94, and Lamin A/C were detected only in total cellular lysates (Supplementary Fig. S2).

### EVs internalization

**Figure 2.**
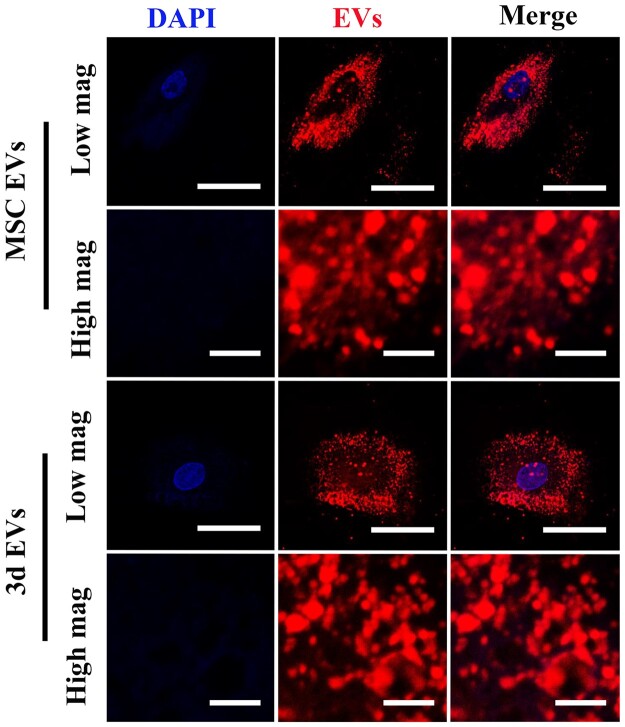
EVs internalization by CLSM. CLSM images of MSC EVs and 3d EVs uptake by hBMSCs. Isolated EVs were prestained with exosome labeling marker (red), then incubated with hBMSCs. Nuclei were stained with DAPI (blue). Representative CLSM images are shown in the figure (*n* = 3). low magnification scale bar: 50 μm. High magnification scale bar: 5 μm.

As important nanocarriers, exosomes must internalize into recipient cells to transfer information. Previous studies indicated that PKH carbocyanine dyes, including PKH67 and PKH26, have been frequently used for staining artificial or biological membranes, including exosomes [36]. In this work, confocal laser scanning microscopy (CLSM) was used to assess the MSCs EVs and 3d EVs internalization into cells. PHK26-labelded MSCs EVs and 3d EVs were incubated with hBMSCs for 12 h on coverslips. Previous study indicated that the uptake of exosomes is time, recipient cells, and temperature dependent. For example, exosomes internalization into HEK 293 cells reached saturation around 12 h following decrease after 24 h [37]. In this study, CLSM images showed that the MSCs EVs and 3d EVs had similar and robust internalization by hBMSCs at 12 h (Fig. 2).

### Surface characterization of EVs hybrid scaffold

**Figure 3.**
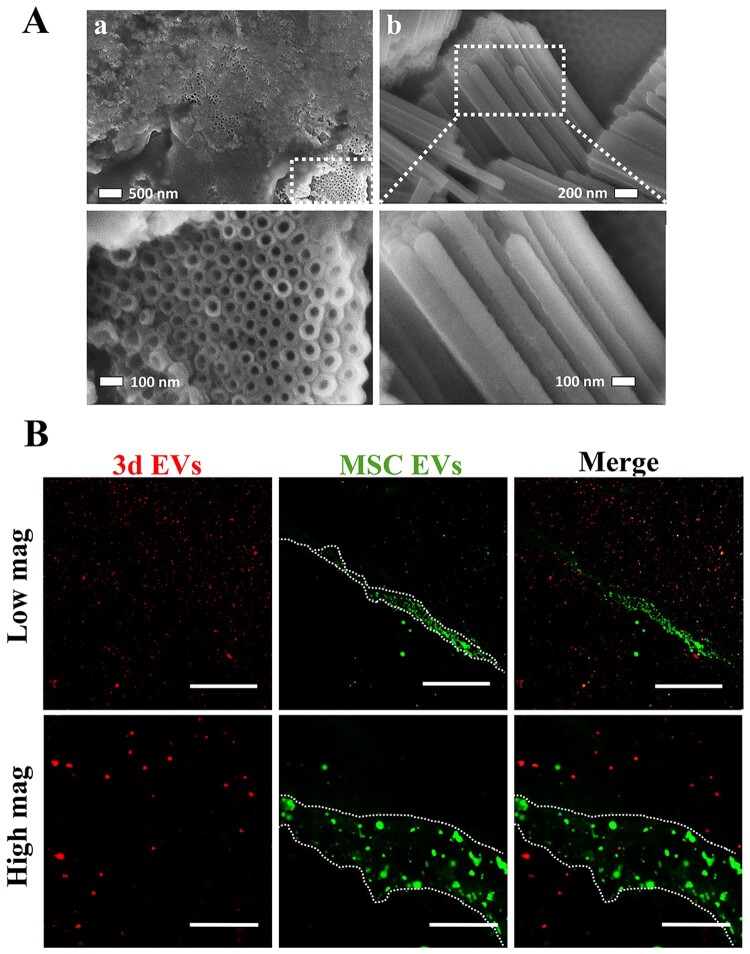
Generation of EV-hybrid TNT. (A) SEM image of anodized TNT after dopamine coating. (A) top-view of the TNT arrays; (B) cross-section of the TNT arrays. In order to expose nanotube structure after dopamine coating and view the cross section, surgical scalpel blade (#11) was used. The SEM image shows nanotube structures, with pore diameters of about 100 nm. (B) Representative CLSM images of TNT/3d EV/MSC EV hybrid scaffolds. MSC EVs were prelabeled with green exosome labeling marker, PKH67, while 3d EVs were prelabeled with red exosome labeling marker, PKH26. To expose MSC EVs coating, a small scratch was generated by using 25-G needle (indicated by dashed white line). Representative CLSM images are shown in the figure (*n* = 3). Scale bar =100 μm (low mag); scale bar =20 μm (high mag).

**Figure 4.**
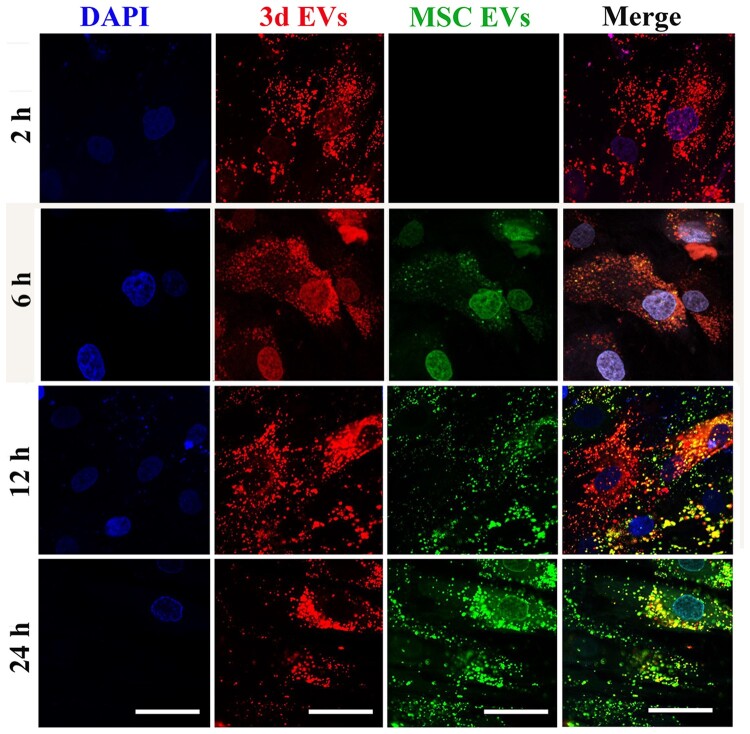
The release of EV from EV-hybrid TNT. MSC EVs were prelabeled with green exosome labeling marker, PKH67, while 3d EVs were prelabeled with red exosome labeling marker, PKH26. EV-hybrid TNT was coculture with hBMSCs. Intense red fluorescence staining was initially uniformly distributed around nuclear areas, while green fluorescence staining was later observed in hBMSCs. The nuclei were stained with DAPI (blue). Representative CLSM images are shown in the figure (*n* = 3). Scale bar =50 μm.

As a simple and one-step surface polymerization method, polydopamine-based grafting layer exhibits high affiliation towards various molecules [38]. Previous study indicated successful immobilization of macrophages-derived exosomes on the surface of polydopamine-modified TNT [17]. Therefore, in this study, TNT was firstly modified by polydopamine. The surface morphology of TNT after poly(dopamine) coating is shown in Fig. 3A. SEM images indicated a self-polymerized poly(dopamine) film was formed on the TNT. The cross-section images revealed that the average diameter of TNT is around 100 nm. To visualize EVs deposition, MSC EVs were firstly labeled with green fluorescence marker, PKH67, followed by incubating with TNT, generating MSC EVs layer, while PHK26-labelded 3d EVs (red) hydrogel layer were deposited on MSC EVs layer, generating TNT/3d EV/MSC EV hybrid. To further expose MSC EVs coating under 3d EVs hydrogel layer, a small scratch was generated using 25-G needle. As shown in Fig. 3B, red punctate fluorescence staining (3d EVs) was uniformly distributed in the hydrogel layer, while green punctate fluorescence staining (MSC EVs) was evident around the scratch area. To further validate EVs release from the hybrid scaffolds, EVs hybrid TNT were incubated with hBMSCs. As shown in Fig. 4, red fluorescence staining (3d EVs) was initially uniformly distributed around nuclear areas at 2 h post-incubation, while green fluorescence staining (MSC EVs) was uptake by hBMSCs at 6 h, and 12 h post-incubation. At 24 h, more green punctate fluorescence staining was visible in hBMSCs.

### The effect of EVs hybrid scaffolds on macrophage viability

**Figure 5.**
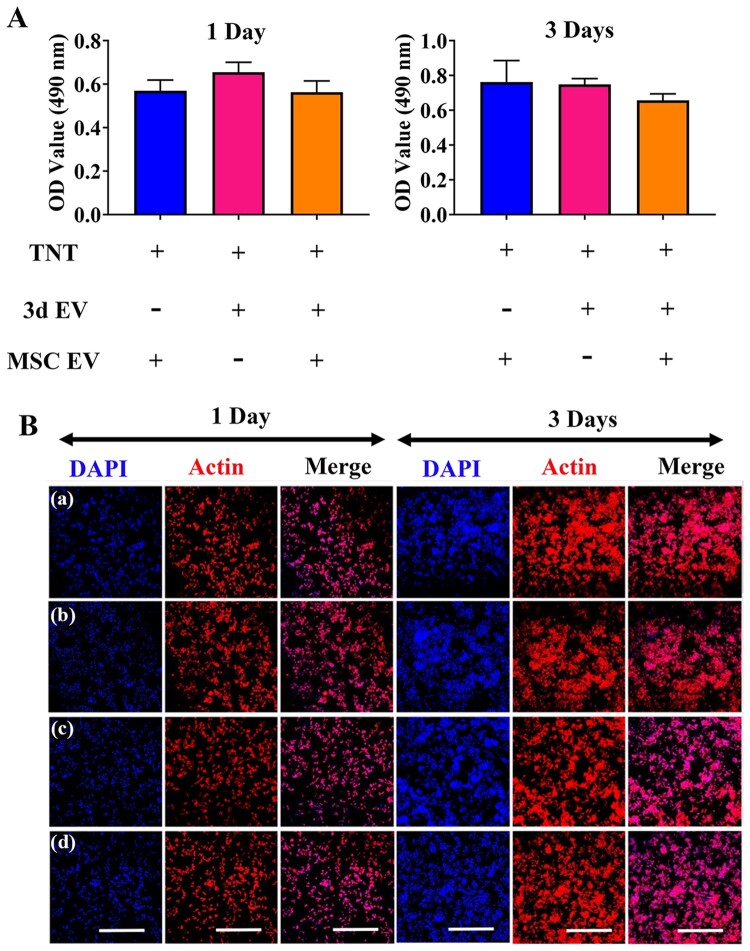
The influence of EV-hybrid TNT on RAW 264.7 and cellular morphology at day 1 and day 3. (A) Cytotoxicity of RAW 264.7 cells on EV-hybrid TNT. The cytotoxicity was determined by MTT assay. No statistically significant difference was observed among the tested groups. (B) CLSM images of macrophages on EV-hybrid TNT. The actin was stained with phalloidin (red), and nuclei were stained with DAPI (blue). Representative CLSM images are shown in the figure (*n* = 3). In (a–d) refer to TNT/MSC EV, TNT/3d EV, TNT/3d EV/MSC EV hybrid, and normal control, respectively. Scale bar =500 μm.

Current knowledge of titanium scaffold impact on macrophage differentiation has been acquired from various titanium-based materials. For example, there was no significant difference of macrophage proliferation between peptide-conjugated titanium implant and titanium control [39]. However, different macrophage adhesion properties and proliferation rate have been recorded when different sized TNT were applied [40]. To assess cell viability in macrophages caused by different EVs scaffolds, cell proliferation was initially measured by MTT assay. As shown in Fig. 5A, no decrease in cell viability was detected from day 1 to day 3. RAW264.7 attached similarly on all EVs scaffolds with no significant difference being observed at 1 day. To further test the cytotoxicity of EVs hybrid scaffolds on macrophages, overall morphology of RAW264.7 were further observed using CLSM (Fig. 5B). Cell seeding on different EVs scaffolds resulted homogeneous cell distribution. Compared with normal control, cells cultured on different EVs scaffolds predominantly adopted a round morphology after 3 days of culture, with gradual increase of cell population, indicating EVs hybrid scaffold have no significant cytotoxicity effect.

### The effect of EVs hybrid scaffolds on inflammatory macrophage polarization

**Figure 6.**
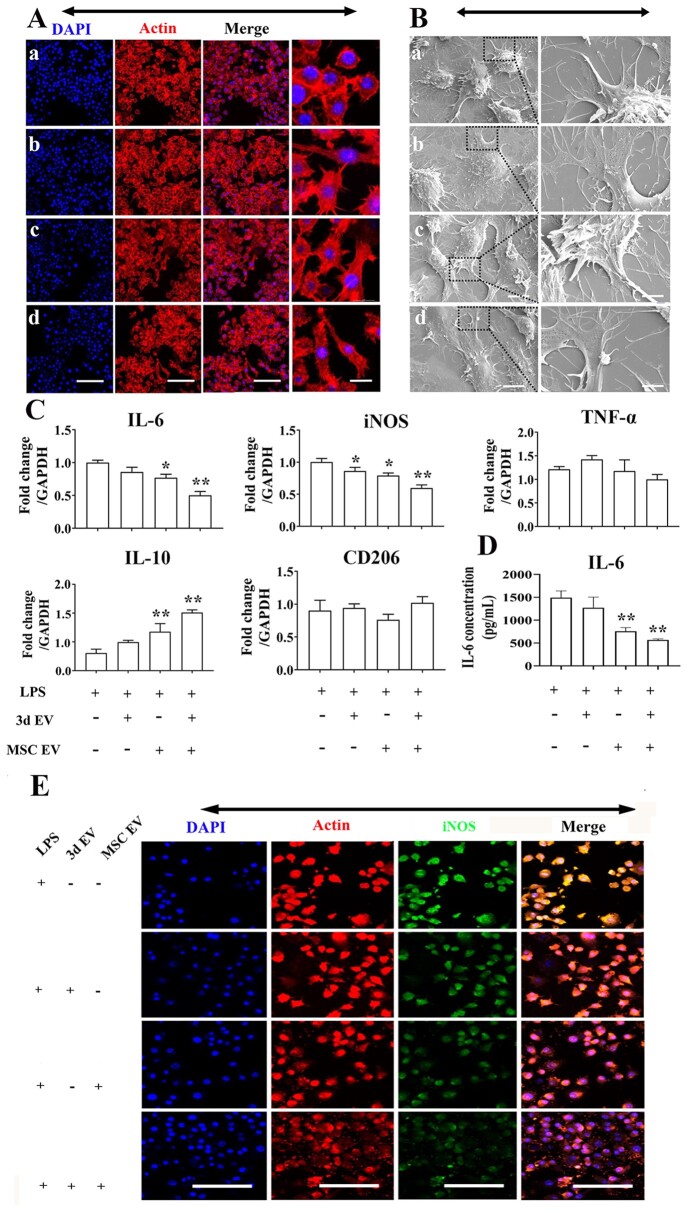
The response of RAW 264.7 to EV-hybrid TNT under inflammatory environment with LPS stimulation. (A) CLSM images of macrophages response to EV-hybrid TNT. Inflammatory environment was induced by 1000 ng/mL LPS stimulation for 6 h. Cells were washed by PBS and cocultured with different EV-hybrid scaffolds. The actin was stained with phalloidin (red), and nuclei were stained with DAPI (blue). Representative CLSM images are shown in the figure (*n* = 3). in (a–d) refer to LPS, 3d EV, MSC EV, and 3d EV/MSC EV hybrid, respectively Scale bar =100 μm (low mag); scale bar =20 μm (high mag). (B) Representative SEM images of macrophages response to EV-hybrid TNT under inflammatory environment. In (a–d) refer to LPS, 3d EV, MSC EV, and 3d EV/MSC EV hybrid, respectively. Scale bar =6 μm (low mag); Scale bar =2 μm (high mag). (C) Relative expression of pro-inflammatory and anti-inflammatory genes in macrophages cocultured with EV-hybrid TNT. The values were normalized to GAPDH as a housekeeping gene. *Indicates statistically significant difference (*P* < 0.05) compared to LPS group. **Indicates statistically significant difference (*P* < 0.01) compared to LPS group. (D) Expression of IL-6 produced in response to EV-hybrid TNT under inflammatory environment. The level of IL-6 was measured using cytokine-specific ELISA. All data are expressed as mean ± SD. **Indicates statistically significant difference (*P* < 0.01) compared to LPS group. (E) CLSM images of iNOS expression. All scale bar: 100 μm.

Bone healing initiated immediately after injury, marked by initial hematoma formation and immune cell infiltration [41]. In the acute inflammatory phase, several key bioactive molecules and immune regulators, such as interleukin 1β (IL-1β), tumor necrosis factor (TNF-α), transforming growth factor-beta (TGF-β) superfamily, and IL-17 are released, primarily by monocyte-derived inflammatory macrophages [42]. Previous studies indicated the importance of macrophages in bone regeneration by blocking their function [43]. In addition, Claudia et al. [43] indicated that pro-inflammatory M1 macrophages are predominant at 24 h post-osteotomy while a gradual infiltration of M2 phenotypes were noted at 3 days post-surgery. Therefore, the potential interaction of EVs hybrid scaffolds on inflammatory macrophages is of particular interest considering the multifaced role of inflammatory macrophages in early bone regeneration. In other scenario, dysregulated or elevated inflammation, as in diabetes, obesity, or aging populations, are often associated with poor bone regeneration [44]. For instance, our previous study indicated significantly elevated IL-1β protein levels in delayed healing model than normal healing model [45]. Therefore, we then tested the regulatory role of EVs hybrid scaffolds on inflammatory macrophages. In this study, CLSM, SEM, gene expression, and ELISA were used to evaluate inflammatory macrophage response to EVs hybrid scaffolds. Addition of LPS, caused cells to change to pancake-like morphology. CLSM images indicated slightly elongated morphology in the EVs hybrid group (Fig. 6A). We further analyzed the cellular morphology in the coculture system using SEM. There was certain cellular elongation when EVs hybrid scaffold were cocultured with inflammatory macrophages (Fig. 6B). We further analyzed the inflammatory and anti-inflammatory genes and IL-6 cytokine expression using RT-PCR and ELISA. There was a significant decrease of inflammation-related genes in the MSCs EVs and EVs hybrid scaffolds. The expression of IL-6 and iNOS in EVs hybrid were 2-fold and 1.8-fold decrease compared to LPS control (Fig. 6C**)**. By comparison, the IL-6 expression was significantly decreased in the EVs hybrid scaffold (Fig. 6D). As shown in Fig. 6E, iNOS-positive staining also demonstrated the same tendency. Recent studies also indicated M2-polarizing potential of MSCs-derived exosome on macrophages. For example, Liu et al. indicated that M2b polarizing potential of MSCs-derived exosomes on colonic macrophage in inflammatory bowel disease [46]. He et al. [47] also found that treatment of macrophages with MSCs-derived exosomes could induce M2 phenotype polarization.

### EVs hybrid scaffolds promotes MSCs migration

**Figure 7.**
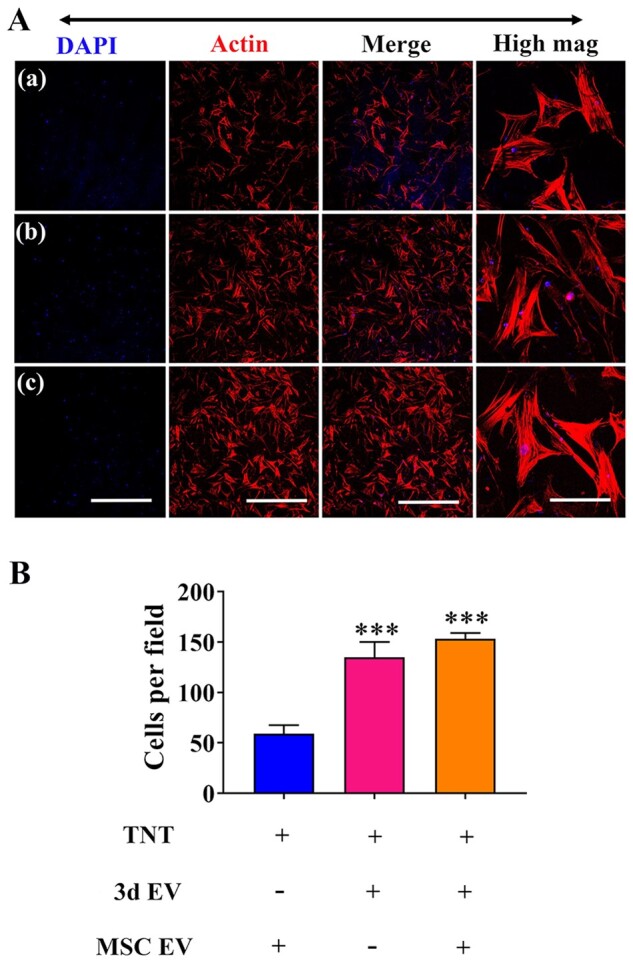
hBSMCs migration induced by EV-hybrid TNT. (A) Transwell migration assay was performed to determine the effect of EV-hybrid TNT on hBMSCs migration. After 12 h, the cells on the upper surface was gently removed, while cells migrated to the beneath membrane were fixed. The actin was stained with phalloidin (red), and nuclei were stained with DAPI (blue). Representative CLSM images are shown in the figure (*n* = 3). In (a–c) refer to TNT/MSC EV hybrid, TNT/3d EV hybrid, and TNT/3d EV/MSC EV hybrid, respectively. Scale bar =500 μm (low mag). (B) Semi-quantification of migrated cells. ***Indicates statistically significant difference (*P* < 0.001) compared to TNT/MSC EV hybrid.

Recruitment of MSCs to the injury site is the incipient step for bone formation [48]. Once recruited under various chemokines or cytokines, MSCs can proliferate and differentiate into osteogenic precursor cells, followed by secretion of extracellular matrix proteins that boost bone formation [49, 50]. Abnormal migration of MSCs are often noted under pathological conditions. For example, there was significant decrease in MSCs migration isolated from ovariectomised (OVX) rats compared to MSCs isolated from adult and young rats [51]. Therefore, therapeutic methods that can stimulate recruitment and homing of MSCs are becoming promising approach for bone regeneration. Migratory ability of MSCs under the influence of scaffolds was investigated by measuring the amount of MSCs migrated to the lower side of transwell membrane. We found there was significantly increase of MSCs migration in EVs hybrid scaffold (Fig. 7A, B). When 3d EVs was added, the mean number of cells that migrated was 150 ± 9 compared to the control. In spite of the MSC EVs immobilization, the mean number of cells that migrated remain unaffected compared to 3d EVs group (Fig. 7B).

### EVs hybrid scaffolds influence the osteogenesis of MSCs

**Figure 8.**
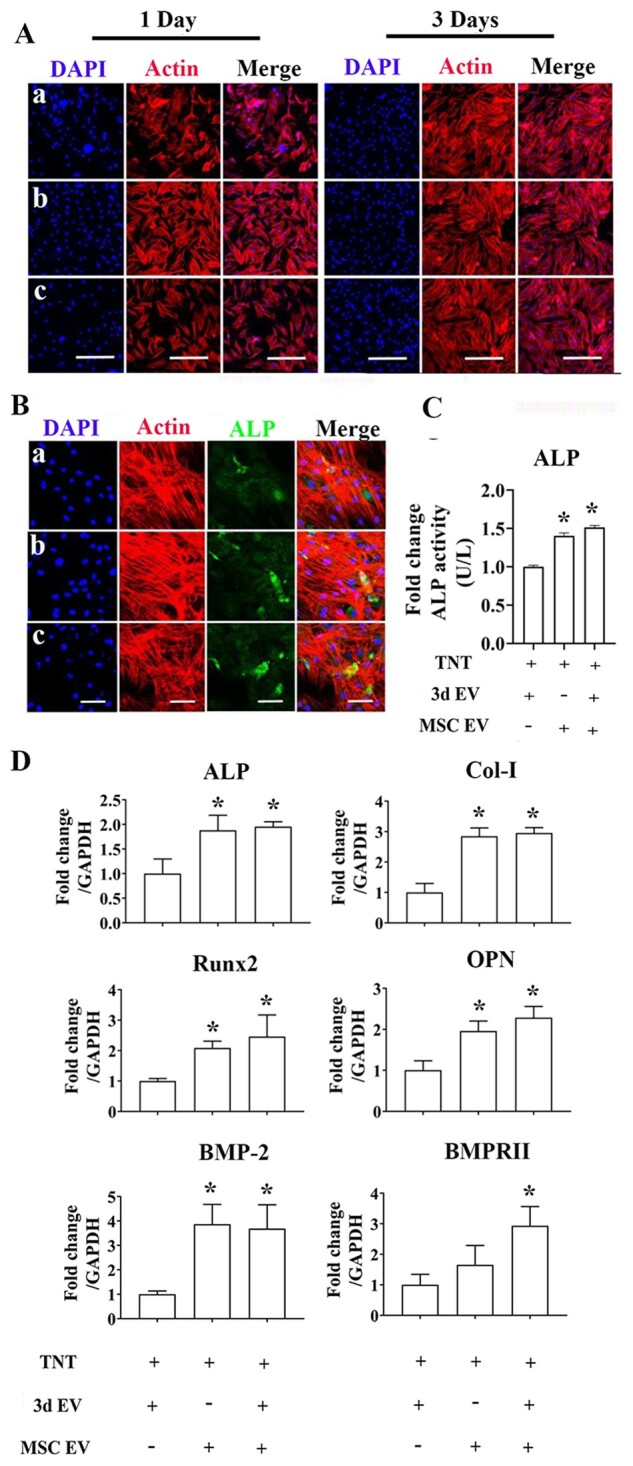
The effect of EV-hybrid TNT on hBMSCs. (A) CLSM images of hBMSCs response to EV-hybrid TNT. In (a–c) refer to TNT/3d EV hybrid, TNT/MSC EV hybrid, and TNT/3d EV/MSC EV hybrid, respectively. The actin was stained with phalloidin (red), and nuclei were stained with DAPI (blue). Representative CLSM images are shown in the figure (*n* = 3). Scale bar = 500 μm. (B) Representative CLSM images of ALP expression. The actin was stained with phalloidin (red), ALP (green), and nuclei were stained with DAPI (blue). Representative CLSM images are shown in the figure (*n* = 3). Scale bar = 100 μm. (C) ALP activity. Error bars denote the mean ± SD (*n* = 3). *Significant difference (*P *<* *0.05). (D) Relative expression of osteogenesis-related genes. Error bars denote the mean ± SD (*n* = 3). Significant differences: *P <* *0.05 compared to TNT/3d EV group.

To test the cytotoxicity of EVs hybrid scaffold on hBMSCs, overall morphology of hBMSCs were further observed using CLSM (Fig. 8A). hBMSCs cytoskeleton staining indicated homogeneous cell distribution. To further investigate the regulatory ability of EVs hybrid scaffold on MSCs differentiation, MSCs were co-cultured with different scaffolds. Gene expressions of osteoblast-specific genes was assessed by RT-PCR, and ALP protein expression was measured by immunofluorescence staining and ALP activity assay. Compared to the TNT/3d EVs, the fluorescence intensity of ALP on day 7 was significantly enhanced in the EVs hybrid group (Fig. 8B). The ALP activity assay results also showed similar trend (Fig. 8C). The expression of osteogenesis-related genes (ALP, Col-I, Runx2, and OPN) were quantitatively measured by RT-PCR (Fig. 8D). mRNA expression further confirmed a significant increase of osteogenesis-related genes in EVs hybrid scaffold than the control. As important nanocarriers, a series of regulatory miRNAs with osteodifferentiation potential have been identified in MSCs-derived exosomes [33]. The mechanisms of the regenerative ability of MSCs-derived exosomes on osteogenesis and bone regeneration may also rely on their roles by targeting various cellular signaling pathways [34]. For examples, phosphoinositide 3-kinase/protein kinase B (PI3K/AKT), mitogen-activated protein kinase, and bone morphogenetic proteins (BMP)/Smad, and Wnt/β-catenin signaling pathways have all been indicated involving this process. BMPs are a group of osteoinductive molecules that play an important role in skeletal development and bone regeneration [52]. Among all the BMPs studied so far, BMP-2 is one of the most important growth factors for bone regeneration. The effect of exosomes isolated from MSCs on BMP-2 signaling pathways have been studied previously. For example, human umbilical cord MSCs-derived exosomes can significantly reduce the pathological changes and apoptosis of the femoral head necrosis via upregulation of BMP-2 and VEGF [53]. A recent study using exosomes isolated from naïve MSCs also demonstrated marked increase of BMP-2 expression, indicating significant pro-osteogenic effect of MSCs-derived exosome [22]. In our study, we further evaluate the regulatory ability of EVs hybrid scaffolds on the osteogenic differentiation of MSCs via BMP-2 signaling pathway. At day 3, the EVs hybrid scaffold demonstrated high expression of BMP-2 and BMPRII compared to the control, indicating possible activation of BMP-2-related signaling pathway (Fig. 8D).

## Conclusions

In this study, we generated a double-layered EVs incorporated TNT to regulate MSCs cellular behaviors as well as potential immune response. The top hydrogel layer incorporated with 3d EVs on TNT functions as MSCs attractant, while the lower polydopamine layers incorporated with MSC EVs serves as pro-osteogenic factor. Our results have shown that this EVs hybrid TNT may represent a novel stem cell-free therapy for implant modification to enhance the migration and osteogenic differentiation of hBMSCs *in vitro*. In the meantime, this double layered EVs incorporated TNT shows immuno-regulatory role for macrophages. However, there are many limitations for this study which need to be addressed in the future. For instance, further studies are needed to assess the activation of BMP-2-related signaling pathways and other signaling crosstalk, especially assessing the long-term effects for this EVs hybrid TNT for bone regeneration. In addition, as a proof of concept, we have not tested this EVs hybrid scaffold in animal model. Therefore, further animal study is warranted to test the functionality of this EVs hybrid scaffold, especially its preservation during and after implantation. Third, this study used murine-derived macrophage cell line instead of human macrophages to understand the immune cells response to EVs hybrid scaffold. In the future, more studies using human macrophages should be performed in order to better understand the macrophage response to EVs hybrid scaffold *in vitro*. Fourth, considering the number of tests needed to be performed for ensuring the aim of the study, we excluded the TNT as control and used EVs modified TNT instead. In the future, TNT with EVs should be incorporated to further validate our result. As exosomes characterization and quantitative analysis on scaffolds are very limited, more methods will be incorporated in the future to validate our concept. In conclusion, this study may represent a pilot test to generate other EVs-hybrid scaffolds (e.g. EVs hybrid pure titanium scaffold) by using exosomes from different sources for better tissue regeneration.

## Author contribution

All authors made substantial contributions to conception and design, acquisition of data, or analysis and interpretation of data; took part in drafting the article or revising it critically for important intellectual content; gave ﬁnal approval of the version to be published; and agree to be accountable for all aspects of the work.

## Supplementary Material

rbab010_Supplementary_Data
