# Supplementary material for: Surface engineering of titania nanotubes incorporated with double-layered extracellular vesicles to modulate inflammation and osteogenesis
Source: Regen Biomater. 2021 May 11;8(3):rbab010. doi: 10.1093/rb/rbab010 (PMC8240597; doi:10.1093/rb/rbab010)
Supplement: rbab010_Supplementary_Data [file rbab010_supplementary_data.docx]

**Supplementary Figure legends**

**Supplementary Figure 1. Analysis of the exosome protein concentration using Pierce™ BCA Protein assay kit**. Error bars denote the mean ± SD (n=3).

**Supplementary Figure 2.** **Characterization of calnexin, Lamin A/C, and Grp94 expression by western blot**. Calnexin, Grp94, and Lamin A/C were used as negative controls.

**Supplementary Figure1**

**
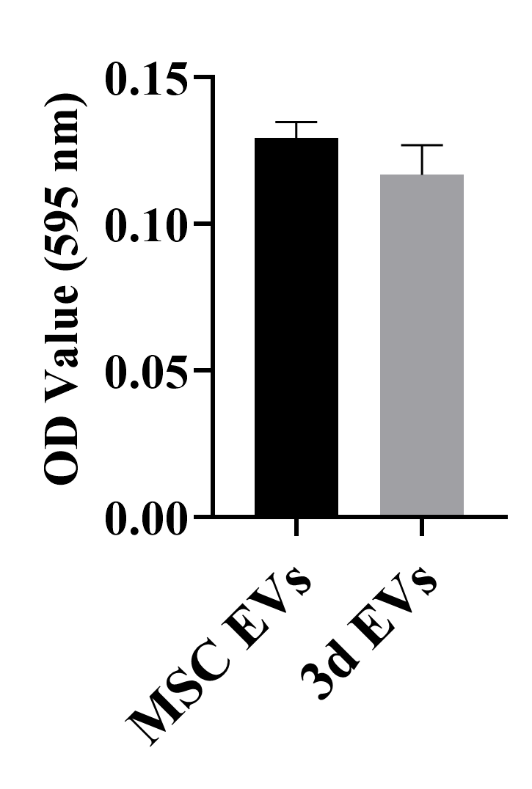
**

**Supplementary Figure2**

**
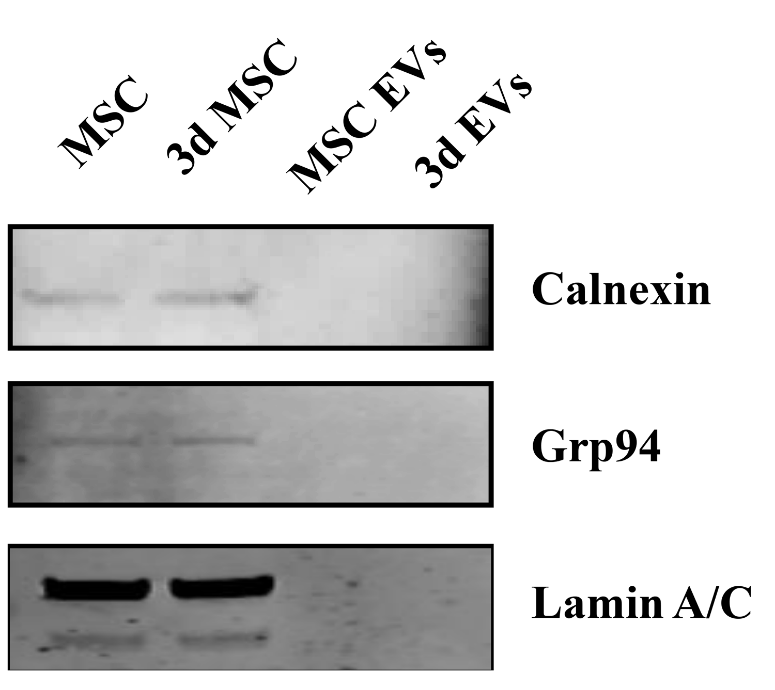
**
